# Sponge-derived Ageladine A affects the *in vivo* fluorescence emission spectra of microalgae

**DOI:** 10.1371/journal.pone.0242464

**Published:** 2020-11-19

**Authors:** Carolin Peter, Silke Thoms, Florian Koch, Franz Josef Sartoris, Ulf Bickmeyer

**Affiliations:** 1 Division of Biosciences, Department of Ecological Chemistry, Alfred Wegener Institute Helmholtz Centre for Polar and Marine Research, Bremerhaven, Germany; 2 Department 2, University of Applied Sciences, Bremerhaven, Germany; 3 Division of Biosciences, Department of Integrative Ecophysiology, Alfred Wegener Institute Helmholtz Centre for Polar and Marine Research, Bremerhaven, Germany; Pondicherry University, INDIA

## Abstract

In several marine hosts of microalgae, fluorescent natural products may play an important role. While the ecological function of these compounds is not well understood, an interaction of these molecules with the photosynthesis of the symbionts has been suggested. In this study, the effect of Ageladine A (Ag A), a pH-dependent fluorophore found in sponges of the genus *Agelas*, on microalgal fluorescence was examined. The spectra showed an accumulation of Ag A within the cells, but with variable impacts on fluorescence. While in two *Synechococcus* strains, fluorescence of phycoerythrin increased significantly, the fluorescence of other *Synechococcus* strains was not affected. In four out of the five eukaryote species examined, chlorophyll *a* (Chl *a*) fluorescence intensity was modulated. In *Tisochrysis lutea*, for example, the position of the fluorescence emission maximum of Chl *a* was shifted. The variety of these effects of Ag A on microalgal fluorescence suggests that fluorophores derived from animals could play a crucial role in shaping the composition of marine host/symbiont systems.

## Introduction

Many sponges harbour photosynthetic symbionts, such as cryptophytes, diatoms, dinoflagellates, and members of the cyanobacterial genus *Synechococcus*, [1–8]. While ultraviolet, violet and blue light penetrate seawater the farthest, longer wavelengths, readily absorbed by typical algal pigments, are attenuated at shallower depths [9]. In 1968, Read et al. [10] discovered fluorophores which, when excited by ultraviolet/blue light, emit blue to green light in demosponges. Schlichter et al. [11, 12] found pigments in the deep-water coral *Leptoseris fragilis* that also transformed light of shorter wavelengths to longer wavelengths. It was proposed that this wavelength-transforming system by the host, enabled photosynthesis under otherwise suboptimal light qualities and intensities. Thus, it may be the case, that only a combination of low light adapted endosymbionts and a light-amplifying mechanism by the host enables phototrophic growth at depths of 95–145 m, where *L*. *fragilis* is typically found [13]. In addition, fluorescing pigments, which change the spectral composition of incoming light to be more suitable for algal photosynthesis, have been found in sponges as well as deep-water corals. While this was hypothesised by Schlichter et al. [11–13] to occur in corals, no empirical studies have been performed.

This principle may also apply to sponges, where light transmission modulation is known for spongin and Ageladine A (Ag A) found in sponges of the genus *Agelas* [14–16]. Ag A is a brominated pyrrole-imidazole alkaloid with different protonated states. While at neutral to slightly alkalic pH of 7–9, the uncharged species dominates, at a pH of 5–7, the first nitrogen becomes protonated. At higher acidity, pH 3–5, a second proton is gained, and the molecule becomes twice positively charged [17]. Ag A is a pH dependent fluorophore, with its fluorescence reaching maximal intensity at pH 3–4. The peak in excitation wavelength is at 370 nm with the emission ranging from 415 nm to 500 nm with a maximum at 415 nm [18], both measured in water. The excitation/emission profile changes with the environment [19]. Its specific function within the sponge holobiont, has not been identified.

While the uncharged form of Ag A is membrane permeable, once protonated it becomes membrane impermeable. This leads to an efficient accumulation of Ag A in acidic cellular compartments, such as thylakoids [17]. In the latter its fluorescence could potentially affect algal photosynthesis, by making a greater proportion of the incident light available to algal pigments. In addition, according to the Förster theory of energy transfer [20], a close proximity of two fluorophores can change the fluorescence characteristics of both [21], with the resulting fluorescence spectrum differing from the assumed additive spectrum. Since many algal pigments are known to be fluorophores, the presence of Ag A could potentially change the fluorescence signature of algal cells in more complex ways than simply yielding an additional Ag A specific fluorescence peak. While Ag A could alter algal photosynthesis in different ways and to differing extents, this has not been investigated before, and potential interactions between Ag A and algal pigments are unknown.

A recent study by Bickmeyer et al. indicates a profound effect of Ag A on the photosynthetic performance of *Synechococcus bacillaris*. Cells which were exposed to UV radiation of 370–380 nm, produced 250% more oxygen when incubated with 30 μM Ag A than cells in the control. This study, however, only examined one algal strain, and whether or not the underlying mechanisms are universal and likely occur in other prokaryotic and eukaryotic microalgae, remains to be tested. Also, the ecological relevance of a host producing fluorophores which then increase the photophysiological efficiency of its endosymbionts, has not been investigated. Since such fluorophores have been found in multiple hosts/symbiont complexes [10, 12], a system turning unusable light into photosynthetically active radiation may be a new and universal currency for symbiosis.

This study investigates whether Ag A enters algal cells and interacts with the fluorescence of different algal species. The presented data suggest that the interaction depends not only on the pigments present in the different algal classes, but also on the chemical environment within the chloroplasts. Unlike previous studies, which hypothesised that the interactions might be universal, results presented here reveal a more heterogenic picture with different effects observed between algal groups and even within the same genus.

## Material and methods

In this study, 4 prokaryote strains and 5 eukaryote species spanning 3 algal phyla were examined. Four cyanobacteria strains were used, belonging to two pigment types differing in the composition of their phycobilisomes (S1 Table) [22]. *Synechococcus* sp. RCC1084 and *S*. *bacillaris* CCMP1333 belong to the green pigment type 1, while *Synechococcus* sp. RCC539 and RCC791 belong to the orange pigment type 3. The five eukaryotes were comprised of three chlorophytes *Chlorogonium elongatum* (by courtesy of B. Seah), *Micrasterias americana* (SAG 151.80) and *Tetraselmis chuii* (SAG 8 6), plus the haptophyte *Tisochrysis lutea (CCAP 927/14)* and cryptophyte *Rhodomonas salina* (K-1487).

### Culture conditions

The marine algae and freshwater algae were cultivated in K-medium [23] and DY-Vm medium (Bigelow Laboratory, National Centre for Marine Algae and Microbiota, East Boothbay, ME, USA), respectively, made from autoclaved, 0.2 μm North Sea water (salinity 34) and 0.1 μm ultrapure water, respectively. All stock cultures were grown in duplicate cell culture flasks (T25, Sarstedt, Nümbrecht, Germany and T75, TPP Techno Plastic Products AG, Trasadingen, Switzerland). The eukaryotes were cultivated at room temperature with a 18:6 h light:dark cycle of 70–80 μmol photons m^-2^ s^-1^. The *T*. *chuii* and *T*. *lutea* cultures were kept on a shaker (KS 130 control, IKA-Werke GmbH & Co. KG, Staufen, Germany) at 100 rpm. The *Synechococcus* cultures were kept under continuous light in a MIR-253 Sanyo incubator (LabX, Midland, ON, Canada) at 25°C and 30 μmol photons m^-2^ s^-1^. The light intensities were measured using a spherical underwater quantum sensor (SPQA) connected to a LI-1000 DataLogger (LI-COR Biosciences GmbH, Bad Homburg, Germany).

### Fluorescence spectra

The fluorescence spectra were determined for cultures in the exponential growth phase after a four-hour incubation period under the respective growth conditions. The chemicals used were dimethyl sulfoxide (DMSO, Carl Roth GmbH + Co. KG, Lichtentanne, Germany), 6 mM ageladine A (Ag A, Marnas Biochemicals GmbH, Bremerhaven, Germany) dissolved in DMSO and 0.5 M sodium bicarbonate (Sigma Aldrich, St. Louis, MO, USA). The concentration of Ag A used was chosen according to the species’ or strain’s tolerance to the substance. For *Synechococcus* sp. RCC1084, *C*. *elongatum*, *R*. *salina* and *T*. *lutea*, a final concentration of 5 μM Ag A was used. For *Synechococcus* sp. RCC539, RCC791, *S*. *bacillaris* CCMP1333 and *M*. *americana* the final concentration was 10 μM Ag A. *T*. *chuii* was incubated with 30 μM Ag A. In order to eliminate potential effects of Ag A´s solvent DMSO on the cells or the fluorescence signal, the controls also had a final DMSO concentration of 0.5% vol. The final concentration of sodium bicarbonate was 2.5 mM.

Wavelength scans were performed under the confocal laser scanning microscope TCS SP5 (Leica, Wetzlar, Germany). The excitation wavelength was set to 405 nm with the measured emission ranging from 420 nm to 700 nm.

For eukaryotes, the fluorescence signal of single cells was isolated and analysed. Due to their small cell size, this was not possible for the *Synechococcus* samples and thus the whole field of view was analysed. The included media background fluorescence exhibited the local maximum observed in all prokaryote samples at 466 nm. Fluorescence emission data was normalised to the highest emission intensity in the controls.

## Results

**Fig 1:**
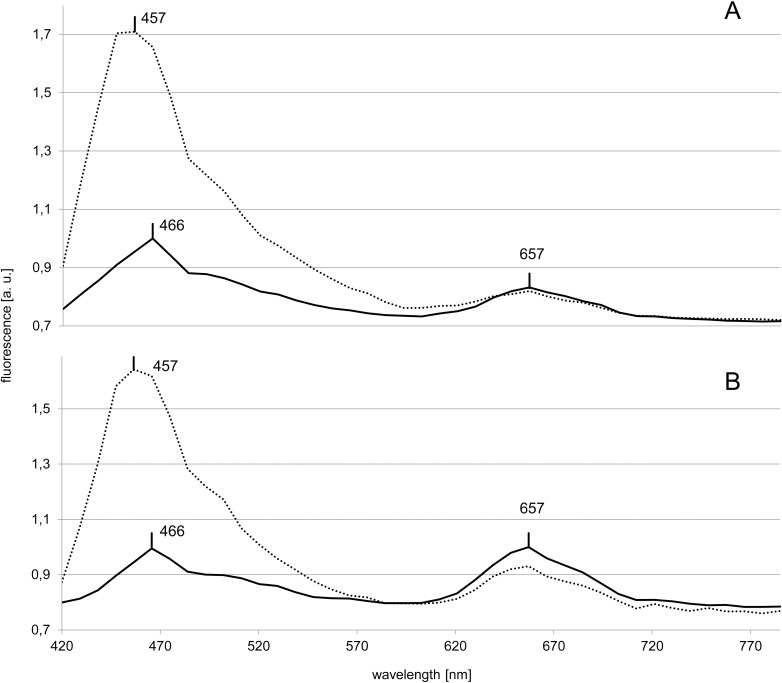
***In vivo* fluorescence spectra of *Synechococcus* sp. RCC1084 (A) and *S*. *bacillaris* CCMP1333 (B).** The solid and dotted lines represent the control and Ag A treatments, respectively. The peak positions are marked at their respective wavelengths.

In the two *Synechococcus* strains belonging to pigment type 1 (*Synechococcus* sp. RCC1084, *S*. *bacillaris* CCMP1333), the controls showed two maxima (Fig 1). One at wavelength 466 nm and a second peak at 657 nm. In the presence of Ag A, the first maximum shifted to 457 nm and increased in amplitude 1.6- to 1.7-fold. Neither the position nor the fluorescence intensity of the second maximum was affected significantly by the addition of Ag A.

**Fig 2:**
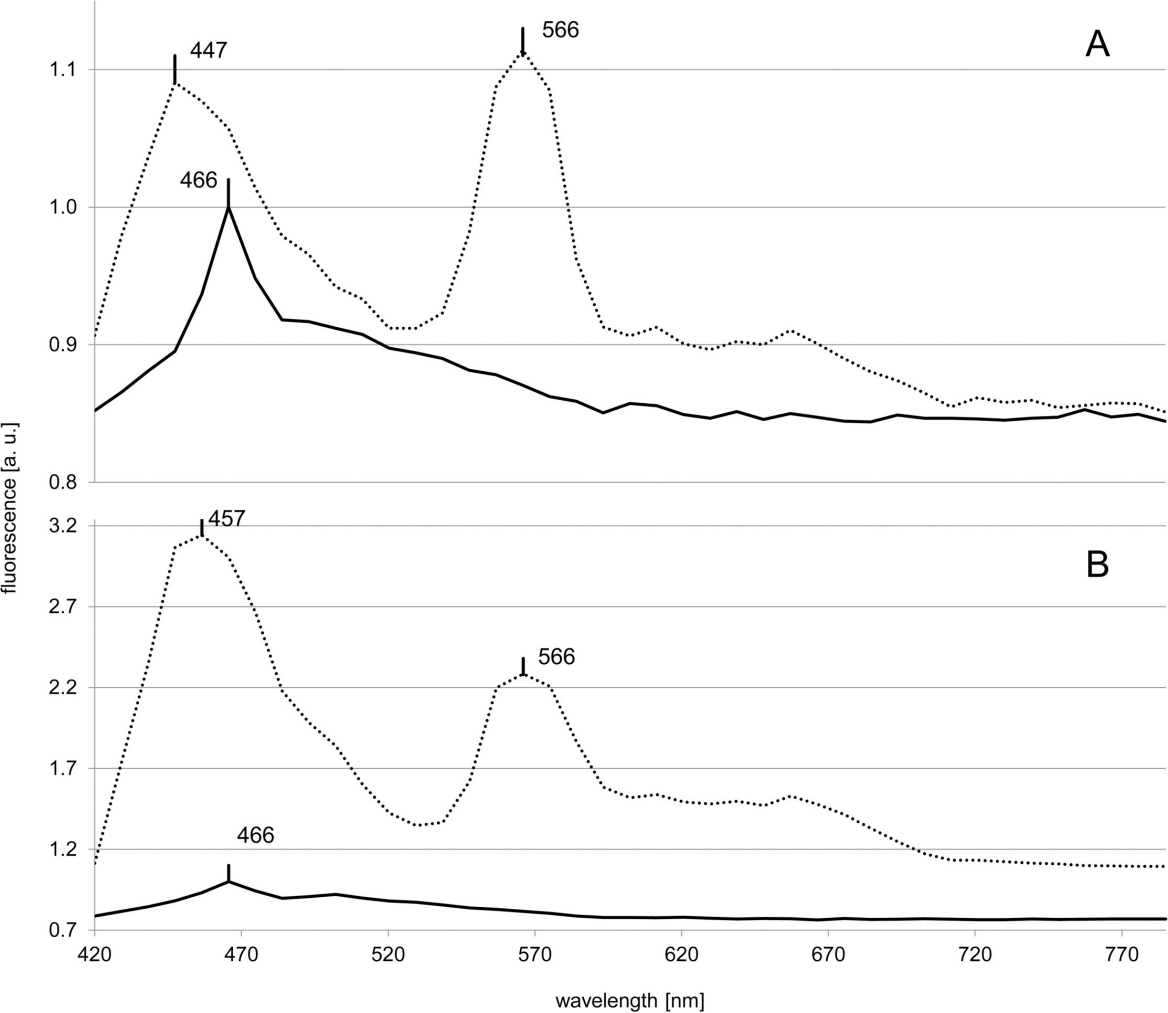
***In vivo* fluorescence spectra of *Synechococcus* sp. RCC539 (A) and *Synechococcus* sp. RCC791 (B).** The solid and dotted lines represent the control and Ag A treatments, respectively. The peak positions are marked at their respective wavelengths.

In the *Synechococcus* sp. strains RCC539 and RCC791, belonging to pigment type 3, the controls exhibited only one maximum at 466 nm (Fig 2). The presence of Ag A shifted the maximum to 447 nm and to 457 nm for RCC539 and RCC791, respectively. While the amplitude of the peak increased 3-fold in RCC791, it did so only by 10% for RCC539. Ag A also led to the formation of an additional maximum at 566 nm in both strains.

**Fig 3:**
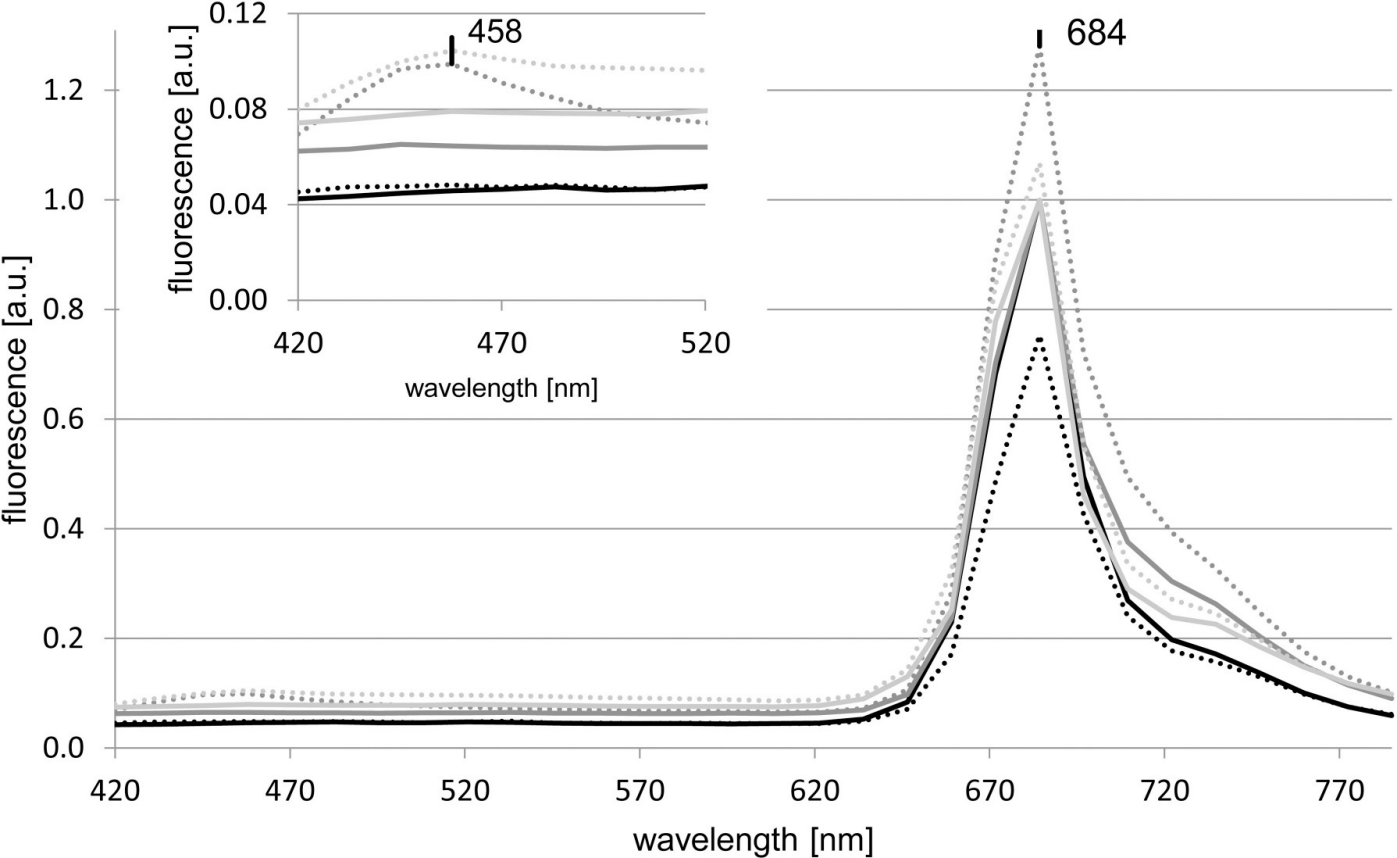
***In vivo* fluorescence spectra of *C*. *elongatum* (black), *M*. *americana* (dark grey) and *T*. *chuii* (light grey).** The solid and dotted lines represent the control and Ag A treatments, respectively. The peak positions are marked at their respective wavelengths.

The three chlorophyte species *C*. *elongatum*, *M*. *americana* and *T*. *chuii* exhibited only one maximum at 684 nm (Fig 3) in the controls. Even though its position was unaffected by Ag A, the amplitude of the peak decreased by 25% in *C*. *elongatum* and increased by 28% in *M*. *americana*. In *M*. *americana* and *T*. *chuii*, the addition of Ag A resulted in an additional, local maximum at 458 nm. *C*. *elongatum* did not show a maximum in that wavelength range.

**Fig 4:**
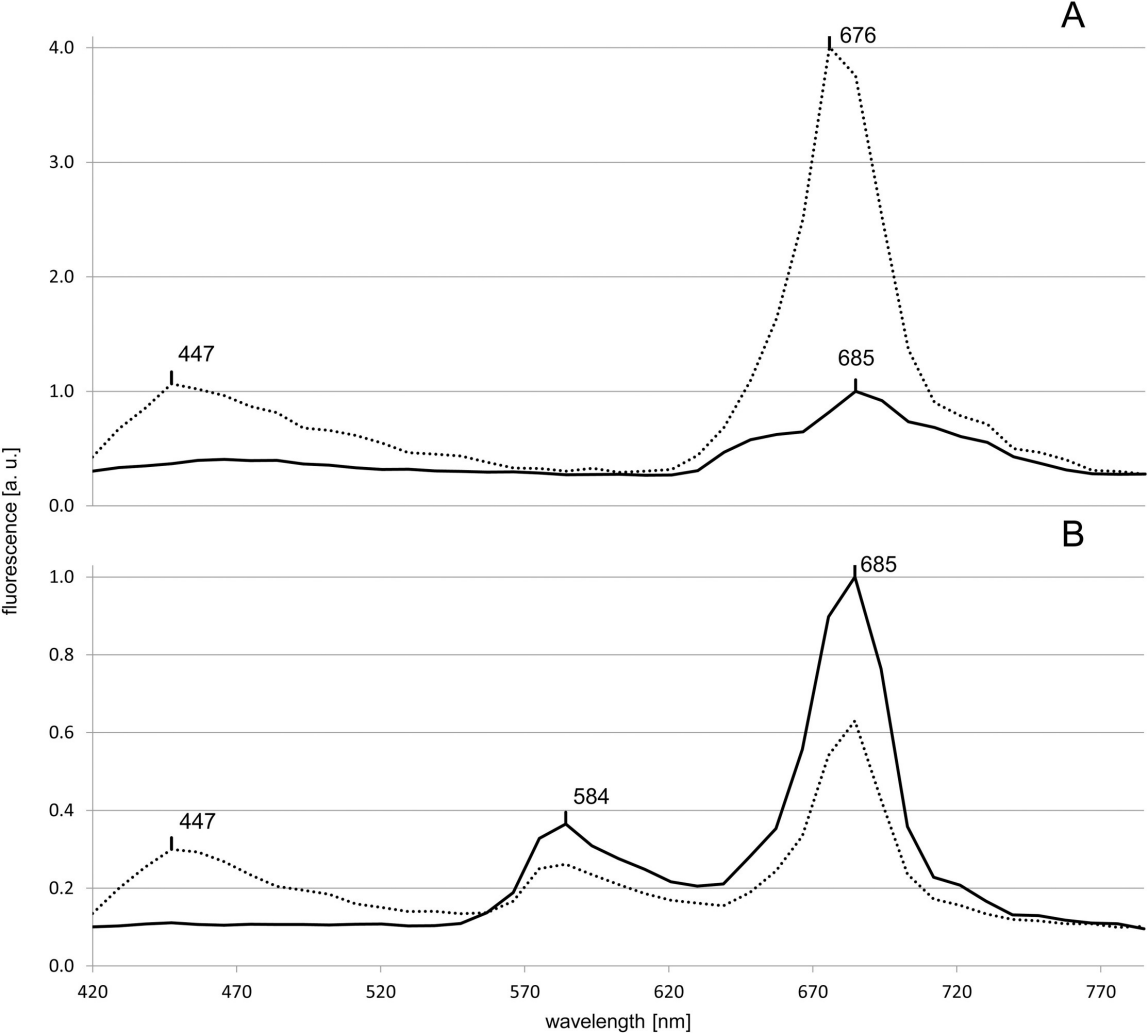
***In vivo* fluorescence spectra of *T*. *lutea* (A) and *R*. *salina* (B).** The solid and dotted lines represent the control and Ag A treatments, respectively. The peak positions are marked at their respective wavelengths.

While in the haptophyte *T*. *lutea*, only one maximum at 685 nm was observed in the control, in *R*. *salina*, a cryptophyte, two maxima, one at 584 nm and one at 685 nm were seen in the control (Fig 4). In the presence of Ag A, both species exhibited an additional maximum at 447 nm. The effect of Ag A on the original maxima differed in that in *T*. *lutea*, it shifted from 685 nm to 676 nm while the fluorescence intensity increased by 300%. In *R*. *salina*, on the other hand, the position of the original maxima remained unaffected, but the amplitudes decreased by 28 and 37%, respectively.

## Discussion

In contrast to initial hypotheses, incubation with Ag A resulted in a variety of effects on algal fluorescence between species, likely caused by differences in the makeup of algal pigments (Table 1).

**Table 1:** Some of the main pigments found in the investigated alga species.

	PC	PE	Chl *a*	Chl *b*	Chl *c*	fucoxanthin
*Synechococcus* sp. RCC539	x	x	x			
*Synechococcus* sp. RCC791	x	x	x			
*Synechococcus* sp. RCC1084	x		x			
*S*. *bacillaris* CCMP1333	x		x			
*C*. *elongatum*			x	x		
*M*. *americana*			x	x		
*T*. *chuii*			x	x		
*T*. *lutea*			x		x	x
*R*. *salina*		x	x		x	

PC and PE stands for phycocyanin and phycoerythrin, respectively while Chl *a*, Chl *b* and Chl *c* stand for chlorophyll *a*, *b* and *c* respectively.

### Fluorescence maxima

#### Ag A

In four out of five eukaryotes, the uptake of Ag A resulted in an additional maximum between 440–460 nm. The maxima were located at 447 nm in *T*. *lutea* and *R*. *salina* and at 458 nm in *M*. *americana* and *T*. *chuii*. This difference in peak position between the algal species as well as its deviance from the characteristics of Ag A in water, may be due to the intracellular conditions such as differences in pH [19]. The only eukaryote which did not exhibit a local maximum in this wavelength range was *C*. *elongatum* (Fig 3). Since it was one of two freshwater chlorophytes tested and the other (*M*. *americana*) exhibited a local maximum, the absence appears to be neither a chlorophyte- nor freshwater specific response. In the *Synechococcus* strains, a combined peak of the Ag A maximum at 447 nm overlapping with the background maximum at 466 nm was observed (S1 Fig). In the strains RCC791, RCC1084 and CCMP1333, this maximum was located at 457 nm, while in *Synechococcus* sp. RCC539 the background maximum merged into the Ag A maximum at 447 nm (Fig 2). As the fluorescence of Ag A is brightest at pH 3 and 4 [18], the difference in fluorescence intensity could be caused by different pH values of the compartments into which it was incorporated.

#### Phycoerythrin

In *R*. *salina*, the maximum at 584 nm (Fig 4), can be attributed to phycoerythrin [24]. The other two algal strains, which also contain phycoerythrin (*Synechococcus* sp. RCC539 and RCC791) showed a maximum (566 nm) attributable to this pigment only in the presence of Ag A (Fig 2). For stress factors like low temperatures [25] and high light intensities [26], significantly increased phycoerythrin fluorescence has been reported to be linked to an uncoupling of the phycobilisomes from the photosystems or the thylakoid membranes. The changes in fluorescence could either be due to Ag A affecting structural components of the phycobilisomes, or specifically the pigments associated with phycoerythrin, phycoerythrobilin (PEB) and phycourobilin (PUB).

In case of *R*. *salina*, only a minor effect of Ag A on phycoerythrin fluorescence intensity was observed and the position of the maximum remained unaffected. Since in cryptophytes phycoerythrin is not organised in phycobilisomes [27], Ag A possibly affects structural components of the phycobilisomes rather than the pigment itself.

#### Phycocyanin

In *Synechococcus* sp. RCC1084 and *S*. *bacillaris* CCMP1333, the maximum at 657 nm (Fig 1) likely belongs to phycocyanin [24]. The fact that in both strains its position and amplitude was largely unaffected by the presence of Ag A, indicates a specific interaction between Ag A and proteins associated with phycoerythrin, present only in the phycobilisomes of *Synechococcus* strains belonging to pigment types 2 and 3. Another possibility is variable interactions between Ag A and different types of phycocyanin (S1 Table).

#### Chlorophyll *a*

A fluorescence maximum, attributable to Chl *a* [28], was observed at 684-685nm in the three chlorophytes and in *T*. *lutea* and *R*. *salina*. The addition of Ag A to *T*. *lutea* shifted the position of the maximum by 9 nm to 676 nm and increased the fluorescence intensity by 300% (Fig 4). In comparison, the increase was significantly lower (28%) in *M*. *americana*, while for *C*. *elongatum* and *R*. *salina*, the fluorescence intensity decreased by 25% and 37%, respectively. This difference in reaction to Ag A could be due to the differences in the molecular composition of the chlorophyll surroundings, including accessory pigments like Fucoxanthin, which is only present in *T*. *lutea*.

### Potential impact on physiology

The additional fluorescence maxima found in all algae except *C*. *elongatum* supports the hypothesis that Ag A is taken up into the cells. The results of this study highlight that Ag A can interact with algal pigments in a multitude of ways. The significant increase in fluorescence of phycoerythrin containing phycobilisomes, for example, indicates a potential disruption in the energy transfer chain for these *Synechococcus* strains. This might decrease their photosynthetic efficiency, while that of strains belonging to pigment type 1 would remain unaffected.

The drastic increase of Chl *a* fluorescence in *T*. *lutea* and *M*. *americana* points to variable effects of Ag A and subsequent changes in photosynthetic activity. However, since photosynthetic efficiency was not assessed in the current study, the effect of Ag A on the former can only be speculated. The increase in fluorescence indicates that an increased proportion of received energy was not being utilised by the photosystems [29]. This could either be because Ag A interrupts the coupling of light harvesting antennae or because Ag A inhibits the photosystems. Given the fine-tuned collocation of absorption maxima within the photosynthetic apparatus, the shift in emission observed in *T*. *lutea* could also inhibit the efficient transfer of energy to the next molecule. However, the increase in fluorescence could also indicate a surplus of light energy made available to the chlorophyll molecules by the fluorescence emission of Ag A. In the latter, a subsequent upregulation of photosystems would be expected, increasing the overall photosynthetic efficiency.

An observed decrease in fluorescence could either hint at decreased or increased photosynthetic efficiency. It could hint at a greater proportion of absorbed light energy being used for photosynthetic activity [29], possibly due to an improved coupling of accessory pigments to the photosystems or by an increased availability of photosystems. On the other hand, decreased fluorescence could also be caused by a decrease in absorption. In this case, reduced photosynthetic efficiency would be expected.

These conflicting effects of Ag A on photosynthetic efficiency need to be investigated with targeted experiments, measuring photosynthesis and other photosynthetic parameters.

## Conclusion

The effects of Ag A on algal pigments are far more complex and diverse than initially proposed by Bickmeyer et al.. While changes in fluorescence intensity could be due to an interaction of Ag A with the photosynthesis apparatus, shifts in fluorescence peak emission suggest a direct pigment-pigment interaction at least for Chl *a*. Since this effect is not observed for all eukaryotic algae, the chemical environment within the chloroplasts also appears to be of great importance. In addition to the interaction with Chl *a*, Ag A appears to have a profound effect on certain phycobilisomes. The differences in the effect of Ag A on algal fluorescence, might enable the sponge to take control over the composition of its microbiome. While in some algae the photosynthetic efficiency would be improved by the presence of sponge produced Ag A, other algae would see their photosynthetic efficiency decreased, placing them at a competitive disadvantage. In symbionts associated with sponges for a prolonged time, this could lead to a change in pigment content or composition, which would enable the algae to thrive where they otherwise could not and to subside on lower resources. It could also serve as a mechanism binding the algae to the sponge. Since fluorophores of similar characteristics as Ag A have also been found in other endosymbiont hosts, this principle might be more widely employed than is currently known.

## Supporting information

S1 Fig Non-normalised fluorescence spectra as determined by wavelength scans of *Synechococcus* sp. RCC1084. The solid line represents the control, the dotted line represents the sample treated with Ag A. The dashed line represents the normalised fluorescence data of *T*. *lutea* treated with Ag A, the dash-dotted line represents the combination peak calculated as the fluorescence in the *Synechococcus* sp. RCC1084 control plus the *T*. *lutea* sample incubated with Ag A.
(DOCX)

S1 Table Composition of phycobilisomes of *Synechococcus* spp. depending on the pigment type. The grey crosses indicate that pigment type 2 has either C-phycocyanin or “R-Phycocyanin II or other”, but not both [22].
(DOCX)

S1 Data (XLSX)
