# Supplementary material for: Sponge-derived Ageladine A affects the in vivo fluorescence emission spectra of microalgae
Source: PLoS One. 2020 Nov 19;15(11):e0242464. doi: 10.1371/journal.pone.0242464 (PMC7676647; doi:10.1371/journal.pone.0242464)
Supplement: S1 Fig — The solid line represents the control, the dotted line represents the sample treated with Ag A. The dashed line represents the normalised fluorescence data of T. lutea treated with Ag A, the dash-dotted line represents the combination peak calculated as the fluorescence in the Synechococcus sp. RCC1084 control plus the T. lutea sample incubated with Ag A. (DOCX) [file pone.0242464.s001.docx]

To verify, that the maximum at 457nm observed in *Synechococcus* sp. RCC1084 samples treated with Ag A is the result of the background maximum at 466nm plus an Ag A maximum at 447nm, a combined peak was calculated. For this, the background maximum of the *Synechococcus* sp. RCC1084 control was added to the Ag A maximum observed in *T. lutea*. Since the concentration of Ag A was assumed to be higher in *T. lutea*, its fluorescence data was normalised by an appropriate factor.
