# Supplementary material for: Sponge-derived Ageladine A affects the in vivo fluorescence emission spectra of microalgae
Source: PLoS One. 2020 Nov 19;15(11):e0242464. doi: 10.1371/journal.pone.0242464 (PMC7676647; doi:10.1371/journal.pone.0242464)
Supplement: S1 Table — The grey crosses indicate that pigment type 2 has either C-phycocyanin or “R-Phycocyanin II or other”, but not both [22]. (DOCX) [file pone.0242464.s002.docx]

| Pigment type | Allophyco-cyanin | C-Phyco-cyanin | R-Phycocyanin II or other | Phyco-erythrin I | Phyco-erythrin II |
| --- | --- | --- | --- | --- | --- |
| 1 | x | x |  |  |  |
| 2 | x | x | x | x |  |
| 3 | x |  | x | x | x |
